# Shift work schedule and damage to nursing workers’ health at Brazilian public hospital

**DOI:** 10.1590/0034-7167-2021-0836

**Published:** 2022-07-18

**Authors:** Rosângela Marion da Silva, Juliana Tamiozzo, Eduardo Rodrigues Lauz, Flávia Camef Dorneles Lenz, Carolina Renz Pretto, Carmem Lúcia Colomé Beck, Alexa Pupiara Flores Coelho Centenaro, José Luís Guedes dos Santos

**Affiliations:** I Federal University of Santa Maria. Santa Maria, Rio Grande do Sul, Brazil; II Federal University of Santa Maria Palmeira das Missões Campus. Palmeira das Missões, Rio Grande do Sul, Brazil; III Federal University of Santa Catarina. Florianópolis, Santa Catarina, Brazil

**Keywords:** Shift Work Schedule, Nursing, Occupational Health, Health, Hospitals, Public., Jornada de Trabalho em Turnos, Enfermagem, Saúde do Trabalhador, Saúde, Hospitais Públicos., Horario de Trabajo por Turnos, Enfermería, Salud Laboral, Salud, Hospitales Públicos.

## Abstract

**Objective:**

to compare work-related damage between the day and night shifts of nursing workers in at a public hospital.

**Methods:**

a cross-sectional, and correlational study, conducted with 308 nursing workers from a Brazilian public hospital. Data collection took place from September 2017 to April 2018 using self-administered questionnaires that investigated socio-occupational variables and the effects of work (Work-Related Damage Assessment Scale). Analysis was descriptive and analytical with a 5% significance level.

**Results:**

during the day shift, the variables that most influenced nursing professionals’ health were body pain, headache, back pain, legs pain, and sleep disorders. When comparing the shifts, it was identified that daytime influences the studied variables more than nighttime. Job tenure was correlated with psychological damage.

**Conclusions:**

the results showed a greater influence of the day shift on the health of professionals who work in a hospital environment. Job tenure influences psychological damage.

## RESUMO

**Objetivo:**

comparar os danos relacionados ao trabalho entre os turnos diurno e noturno de trabalhadores de enfermagem de um hospital público.

**Método:**

estudo transversal, correlacional, realizado com 308 trabalhadores de enfermagem de um hospital público brasileiro. A coleta de dados ocorreu de setembro de 2017 a abril de 2018. Utilizou-se questionários autoaplicáveis que investigaram variáveis sociolaborais e efeitos do trabalho (Escala de Avaliação dos Danos Relacionados ao Trabalho). Análise foi descritiva e analítica, com nível de significância de 5%.

**Resultados:**

no turno diurno, as variáveis que mais influenciaram a saúde dos trabalhadores foram dores no corpo, cabeça, costas, pernas e alterações no sono. Ao comparar os turnos, identificou-se maior influência do diurno nas variáveis estudadas. O tempo de trabalho correlacionou-se com os danos psicológicos.

**Conclusão:**

os resultados mostraram maior influência do turno diurno na saúde dos trabalhadores de enfermagem de ambiente hospitalar. O tempo de trabalho influenciou os danos psicológicos.

## RESUMEN

**Objetivo:**

comparar los daños relacionados con el trabajo entre los turnos diurno y nocturno de trabajadores de enfermería en un hospital público.

**Método:**

estudio transversal y correlacional, realizado con 308 trabajadores de enfermería de un hospital público brasileño. La recolección de datos ocurrió de septiembre de 2017 a abril de 2018 a través de cuestionarios autoadministrados que investigaron variables sociolaborales y efectos del trabajo (Escala de Evaluación de Lesiones Relacionadas con el Trabajo). El análisis fue descriptivo y analítico, con un nivel de significancia del 5%.

**Resultados:**

en el turno diurno, las variables que más influyeron en la salud de los profesionales de enfermería fueron el dolor en el cuerpo, cabeza, espalda, piernas y alteraciones en el sueño. Al comparar los turnos, se identificó que el día influye más que la noche en las variables estudiadas. El tiempo de trabajo se correlacionó con el daño psicológico.

**Conclusión:**

los resultados mostraron una mayor influencia del turno diurno en la salud de los profesionales de enfermería en ambiente hospitalario. El tiempo de trabajo influyó en el daño psicológico.

## INTRODUCTION

The advance of the neoliberal model has accelerated the precarious work process, especially in health work, which is subject to outsourcing, fragile employment relationships, and few guaranteed rights for health teams, especially nurses^(1-2)^. In the public health system, the negative impacts of this process are reflected in the reduction of workers’ wages and rights and an increase in the workload for them, to reduce costs by hiring more professionals^(2-3)^.

In nursing, the main results of this process are the need to work double shifts due to social and economic vulnerability, and work overload, which damage the health of these workers^(3-4)^. Work-related damage is any damage whose origin may be associated with the work environment, conditions, or processes that affect workers’ physical, mental or social integrity. Physical damage is body aches and biological disturbances. Psychological damage is the negative feelings about oneself and life in general. Social damage is the difficulties in family and social relationships^(5)^.

Nursing workers of public hospitals in Brazil are exposed to damage resulting from working conditions^(6)^ and shift work organization. Working in shifts suggests psychological problems and circadian disruptions^(7-8)^. Added to this, there are physical and mental health problems caused by the exercise of the profession, such as body aches, headache, sleep disorders, minor psychological disorders, and emotional exhaustion^(9-13)^. In addition to the damage caused to the nursing staff, these damages have direct consequences on the quality of care provided, affecting the safety of patients^(11)^ and professionals. As a result, there is an increase in the expenses of health institutions, especially with the removal of professionals, an extension of hospitalizations, and costs with materials^(3,14)^.

In this sense, is important to identify the work-related damage that affects the nursing staff, mitigating the effects on these professionals’ health and contributing data that can strengthen preventive actions.

## OBJECTIVE

To compare work-related damage between the day and night shifts of nursing workers at a public hospital.

## METHODS

### Ethical Aspects

This study was approved by the Research Ethics Committee of the *Universidade Federal de Santa Maria*, Brazil, and is in accordance with Brazilian legislation governing research with human beings (Resolution 466/2012). Participants were instructed about the research objectives, willingness to participate and other ethical aspects involving research with human beings, with their consent expressed by signing the Informed Consent Form (ICF).

### Study design, period and place

The study follows the EQUATOR Network guidelines, using the Strengthening the Report of Observational Studies in Epidemiology (STROBE)^(15)^.

The study took place in a public hospital, managed by the federal government, located in southern Brazil. This institution has 403 inpatient beds with exclusively public funding for their care. Data collection was performed in the adult and child emergency room, medical and surgical inpatient units, adult and child intensive care units, and surgical center unit (surgical center and post-anesthetic recovery). It took place from September 2017 to April 2018.

### Population, sample, inclusion and exclusion criteria

At the time of data collection, the institution had 960 nursing professionals (333 nurses, 500 nursing technicians, and 127 nursing assistants). This categorization reflects the workforce specified in the Brazilian professional legislation. For sample calculation purposes, we considered a 95% confidence level and a 5% sampling error, and the application of these parameters produced a representative minimum sample size of 277 nursing workers. The sample was stratified randomly and determined by professional category considering the finite population.

The inclusion criteria were to act in direct care for patients in the selected units, regardless of the activity shift (day or night). We excluded those on vacation or leave of any kind during the period of data collection.

### Study protocol

Data collection had the collaboration of volunteers (two), undergraduate (three), graduate (one), and scientific initiation scholarship (one) students, who were trained in face-to-face meetings with the research coordinator. They received the collector’s manual with project data and questionnaires.

Participants were invited individually and in their workplace. The ethical issues that govern research with human beings such as anonymity and the voluntary nature of participation were presented to them. Afterward, the data collection questionnaires were delivered, and a return date was set. We considered a loss after the 5^th^ attempt to collect the instruments.

We used a questionnaire for socio-occupational characterization and the Work-Related Damage Assessment Scale (EADRT)^(5)^, all of which were self-administered. The first was constituted by the variables as follows: age; sex; children; professional category; work shift; job tenure ; choice of work shift; marital status; other jobs.

The EADRT was created in 2003 and validated in 2006 in Brazil, and is part of the Inventory on Work and Risks of Illness. The psychometric validation of the inventory was performed based on the factor analysis technique. It is an interdependent scale that assesses work-related damage, and the version published in 2007 was used. It has 29 items, distributed on a Likert-type scale in which: 0 = not once; 1 = once; 2 = twice; 3 = three times; 4 = four times; 5 = five times; 6 = six times or more. The items are grouped into three factors: physical damage (12 items); psychological damage (10 items); and social damage (07 items).

### Analysis of results, and statistics

In the EADRT analysis, the average result of each factor was classified into four levels: above 4.0 (presence of occupational diseases); between 4.0 and 3.1 (severe assessment); between 3.0 and 2.0 (critical assessment); below 1.99 (supportable assessment)^(5)^. Data were double entered and statistically analyzed using the PSS (Predictive Analytics Software, SPSS INc., Chicago, USA), version 18.0 for Windows.

We assessed categorical variables using absolute (n) and relative (%) frequencies. The numerical ones are presented as medians and interquartile range (IQR) for non-parametric data and as mean ± standard deviation (SD) for parametric data. The EADRT reliability was assessed by performing the analysis of internal consistency using Cronbach’s alpha coefficient (0.93). Afterwards, a normality test was performed for numerical variables, and non-parametric tests were adopted, such as the comparison test for two independent samples, for the Mann-Whitney U test. Correlation analyzes followed analysis recommendations^(16)^, and were verified using Spearman’s correlation coefficient. In all analyses, a 5% significance level (p<0.05) was used.

## RESULTS

From the population of 960 nursing professionals, a sample calculation stratified by professional category (277 potentially eligible) was performed. It is noteworthy that 350 professionals and nursing professionals were invited, and that there were 42 losses (10 refusals and 32 questionnaires were not returned). When returning the questionnaires, checking the complete completion of the items ensured that there were no missing data on the variables of interest.

The study sample consisted of 308 workers, 32.5% of which were nurses (n=100), 55.5% were nursing technicians (n=171) and 12.0% (n=37) were nursing assistants.

A percentage of 54.9% (n=169) worked in the day shift, 82.5% (n=254) opted for the work shift and 12.3% (n=38) had another job.

The average age of participants in the day shift was 39.4 years old (±8.58), and in the night shift, 42.6 years old (±9.47). Day shift workers had an average job tenure of 6.68 years (±7.84), and night shift workers, 9.76 years (±8.11). A statistical difference was identified when comparing the shifts in age (p=0.002) and job tenure (p<0.001) (Mann-Whitney U test).

**Figure 1:**
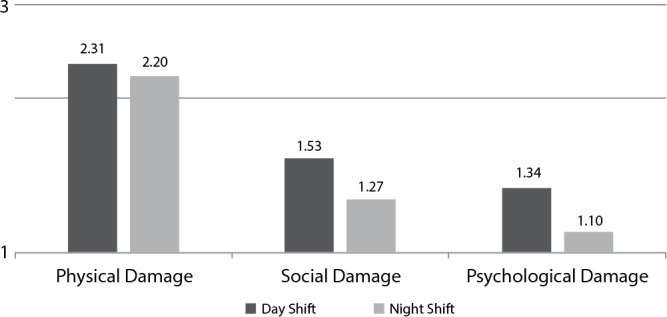
Average of Work-Related Damage Assessment Scale (EADRT) factors in work shifts in a public hospital, Public Hospital, Brazil, 2017-2018

In the averages of the scale factors distributed in day and night shifts, we observed that physical damage had the highest average in day and night shifts, with a critical rating. Psychological damage and social damage presented a bearable classification. There was no statistical difference between scale factors and work shifts (p>0.05) (Figure 1).

In comparison of physical damage items according to work shift, a statistical difference was identified in headache and day shift (p<0.05). No significant relationships were identified in the other items; however, higher averages can be observed in the day shift (Table 1).

**Table 1:** Comparison of physical damage items and day and night shifts, public hospital, Brazil, 2017-2018 (N=308)

**Physical damages**	**DAY** **Mean(±SD)** **Md(IQR)**	**NIGHT** **Mean(±SD)** **Md(IQR)**	*p* value^*^
Body pain	3.57(2.00)	3.36(2.03)	0.393
	4.00(2.00-6.00)	3.00(2.00-6.00)	
Arm pain	2.70(2.11)	2.58(2.03)	0.724
	2.00(1.00-5.00)	2.00(1.00-4.00)	
Headache	3.09(2.14)	2.45(2.14)	0.009
	3.00(1.00-5.00)	2.00(1.00-4.00)	
Respiratory disorders	1.00(1.64)	0.88(1.41)	0.881
	0.00(0.00-2.00)	0.00(0.00-1.00)	
Digestive disorders	1.73(1.79)	1.88(1.82)	0.448
	1.00(0.00-3.00)	2.00(0.00-3.00)	
Back pains	3.82(2.03)	3.43(2.21)	0.098
	4.00(2.00-6.00)	4.00(1.00-6.00)	
Hearing disorders	0.48(1.17)	0.52(1.17)	0.843
	0.00(0.00-0.00)	0.00(0.00-0.00)	
Appetite changes	1.90(2.10)	1.86(2.11)	0.779
	1.00(0.00-3.00)	1.00(0.00-3.00)	
Vision disorders	1.22(1.83)	1.48(1.89)	0.112
	0.00(0.00-2.00)	1.00(0.00-3.00)	
Sleep disorders	3.02(2.21)	3.02(2.28)	0.869
	3.00(1.00-6.00)	3.00(1.00-5.00)	
Leg pain	3.78(1.85)	3.57(2.09)	0.511
	4.00(2.00-6.00)	4.00(2.00-6.00)	
Circulatory disorders	1.50(1.92)	1.30(1.89)	0.198
	1.00(0.00-3.00)	0.00(0.00-2.00)	

* Mann-Whitney U test; SD - standard deviation; Md - median; IQR - interquartile range.

In comparison of social damage items and day and night shifts. There is a significant relationship between work shift and willingness to be alone (p<0.05) (Table 2).

**Table 2:** Comparison of social damage items and day and night shifts, public hospital, Brazil, 2017-2018 (N=308)

**Social damages**	**DAY** **Mean(±SD)** **Md(IQR)**	**NIGHT** **Mean(±SD)** **Md(IQR)**	*p* value^*^
Insensitivity to co-workers	1.47(1.72)	1.25(1.55)	0.328
	1.00(1.00-3.00)	1.00(1.00-2.00)	
Difficulties in relationships	1.30(1.64)	1.07(1.49)	0.129
	1.00(0.00-2.00)	0.00(0.00-2.00)	
Willingness to be alone	2.39(1.98)	1.90(1.94)	0.019
	2.00(1.00-4.00)	1.00(0.00-3.00)	
Conflicts in family relationships	1.62(1.73)	1.31(1.52)	0.126
	1.00(0.00-3.00)	1.00(0.00-2.00)	
Aggressiveness towards others	1.20(1.49)	1.02(1.27)	0.448
	1.00(0.00-2.00)	1.00(0.00-2.00)	
Difficulty with friends	0.85(1.17)	0.71(1.17)	0.318
	0.00(0.00-1.00)	0.00(0.00-1.00)	
Impatience with people in general	1.92(1.77)	1.62(1.56)	0.199
	1.00(1.00-3.00)	1.00(0.00-3.00)	

* Mann-Whitney U test; SD - standard deviation; Md - median; IQR - interquartile range.

In comparison of psychological damage items and day and night shifts no significant relationships were found (p>0.05) (Table 3).

**Table 3:** Comparison of psychological damage items and day and night shifts, public hospital, Brazil, 2017-2018 (N= 308)

**Psychological damages**	**DAY** **Mean(±SD)** **Md(IQR)**	**NIGHT** **Mean(±SD)** **Md(IQR)**	***p*** value^*^
Bitterness	0.90(1.50)	0.85(1.34)	0.887
	0.00(0.00-1.00)	0.00(0.00-2.00)	
Feeling of emptiness	1.27(1.68)	1.13(1.59)	0.410
	1.00(0.00-2.00)	0.00(0.00-2.00)	
Feeling of helplessness	1.23(1.78)	1.04(1.53)	0.470
	0.00(0.00-2.00)	0.00(0.00-2.00)	
Bad mood	1.91(1.78)	1.66(1.60)	0.278
	1.00(0.00-3.00)	1.00(0.00-2.00)	
Willingness to give up everything	1.26(1.80)	0.94(1.55)	0.065
	1.00(0.00-2.00)	1.00(0.00-1.00)	
Sadness	1.71(1.75)	1.40(1.68)	0.061
	1.00(0.00-3.00)	1.00(0.00-2.00)	
Irritation at all	1.71(1.80)	1.38(1.60)	0.119
	1.00(0.00-3.00)	1.00(0.00-2.00)	
Feeling of abandonment	1.16(1.68)	0.88(1.41)	0.125
	1.00(0.00-2.00)	0.00(0.00-2.00)	
Doubt about the ability to do the tasks	1,06(1.38)	0.85(1.29)	0.078
	1.00(0.00-2.00)	0.00(0.00-1.00)	
Loneliness	1.19(1.69)	0.94(1.54)	0.118
	0.00(0.00-2.00)	0.00(0.00-1.00)	

* Mann-Whitney U test; SD - standard deviation; Md - median; IQR - interquartile range.

Health-related situations in terms of physical, psychological, and social damage, regardless of the work shift, were all correlated with each other (Spearman’s correlation coefficient). Other significant and direct correlations were identified between age and vision disorders (r=0.238), circulatory disorders (r=0.199), bitterness (r=0.137), feeling of emptiness (r=0.169), and feeling of abandonment (r=0.116). We also found significant and indirect correlations between age and headache (r= - 0.135) and bad mood (r= - 0.188) items.

By correlating job tenure and variables (Spearman’s correlation coefficient), significant and direct correlations were identified with vision disorders (r=0.196), bitterness (r=0.176), feeling of helplessness (r=0.186) sadness (r=0.131), irritation (r=0.137), loneliness (r=0.149), and psychological damage (r=0.122).

When assessing by work shift, significant and direct correlations were identified in the day shift between age and vision disorders (r=0.297) and circulatory disorders (r=0.260).

By correlating job tenure and variables (Spearman’s correlation coefficient), significant and direct correlations were identified in the day shift with feeling of helplessness (r=0.205) and sadness (r=0.213). And in the night shift, significant and direct correlation with vision disorders (r=0.248).

## DISCUSSION

The results showed a prevalence of physical damage in nursing workers in the day shift of a public hospital, which suggests that this work shift has a greater negative impact on the items assessed than in the night shift. This data differs from the result of a research carried out in Iran with emergency room nurses, which found a significant association between night work and the increased prevalence of physical symptoms of pain^(17)^.

The leg pain, back pain, and body pain items, from the physical damage factor, in day and night shifts, had the highest means, with severe assessment, which indicates a negative result and producing suffering at work^(5)^. These data are consistent with other studies that also identified a high frequency of back, lower and upper limb back pain in hospital nurses^(10,17-18)^.

In Brazil, research carried out with the nursing staff identified a high prevalence of pain or discomfort in the lumbar region, associated with overload, bad mood, fatigue, unsatisfactory work environment conditions, in the shoulder, cervical and hip region^(19)^, and higher occurrence of absenteeism in workers with symptoms of lower back, shoulder, and elbow pain^(20)^.

The nursing work routine in the researched institution differs in work shifts. In the day shift, there is a routine to perform or assist in body hygiene by aspersion or in bed, assisting in walking and forwarding for diagnostic tests and surgical procedures. In the night shift, there is a concern with preparing patients for exams/surgeries, among other care activities. Thus, the highest averages of body, back, leg, and arm pain identified in the day shift are justified. Arm pain, for instance, is commonly reported by nursing workers in scientific literature^(10,18,21)^, and this study presented a critical assessment, producing suffering.

The prevalence of pain in nursing professionals is a constant in research on workers’ health. The research identified a prevalence of elbow, wrist, and/or hand and shoulder pain. Shoulder pain has been associated with reduced concentration at work and the manifestation of presenteeism, suggesting a relationship between back pain and presenteeism^(21)^.

Headaches had a severe assessment during the day shift, suggesting that workers are ill, in line with other studies^(9-10,18)^. Work is referred to as the cause of headaches in hospital care nurses, with a significant association between headaches and poor quality of life in the work environment^(9)^.

The research carried out in Taiwan with hospital nurses found that headaches contributed to developing emotional exhaustion, which influences the intention to give up the profession^(22)^. A study carried out in Norway reported a high prevalence of headaches in nurses with sleep disorders, pointing to the need for more research to explore the associations between headaches and work^(23)^.

Sleep disorders showed similar results between work shifts, with severe assessment, a result that agreed with other research on the topic^(9-10,18)^. Sleep quality was impaired by Spanish nurses’ shift work^(24)^, with an impact on quality of care. On the other hand, another study showed that night shift workers have more issues functioning during the day and more difficulty falling asleep^(25)^.

Psychological damage showed a direct and significant correlation with job tenure, which suggests that the longer the time of work of nursing professionals in the service, the greater the influence of psychological damage on health. Authors state that health workers are more prone to psychological damage due to exposure to violence in the work environment, insecurity and severe psychological demands, conflict with co-workers and workload^(12-13,20)^. Also, the identification of bearable classification and the lowest averages in day and night shifts may be due to the subjectivity of EADRT questions, maybe influenced by individuals’ mood at the time of data collection^(10)^.

Social damage was classified as bearable in both shifts, and only willingness to be alone presented a critical assessment. Regarding this result, literature is not unanimous, and studies that use EADRT have identified a bearable assessment being identified^(10)^ and a critical assessment^(18)^. These discrepancies can be explained by the different nursing care scenarios investigated. In this research, the sample consisted of nursing professionals from different hospital sectors, which may explain the critical assessment in this item. Moreover, factors external to the work environment, such as housework and family relationships, can influence this variable^(18)^.

The bearable assessment, identified in psychological and social damage, is a positive result and produces pleasure at work^(5)^, and is in agreement with other studies^(10,18)^. The prevalence of the highest averages in the day shift in physical and social damage contrasts with recent research data that showed higher rates of physical and psychological risks among nurses who worked the night shift compared to the day shift^(26-27)^.

We need to consider that workers’ health is influenced by work organization, with shift work being an aspect that can contribute to workers’ illness. Nursing workers, as they have to work irregular shifts between night and day, are more susceptible to constant interruptions in biological clocks. Those who work at night, particularly, must forcefully interrupt their biological clocks and, consequently, present a constant state of sleep deprivation^(7)^.

Unlike male nursing professionals, female nurses who work in shifts tend to be more susceptible to developing psychological problems possibly due to biological characteristics and hormones^(8)^. Thus, the need to make the reality of nursing workers’ health visible is justified to open up new possibilities for studies and policies that promote health for nursing professionals.

### Study limitations

The limitations of this research are related to its cross-sectional design, the impossibility of causal inference and the influence of health status at the time of data collection, which may not represent the daily life experienced by the study participants. Despite these limitations, the research characterizes the effects of working in a hospital environment on nurses’ health, pointing out health problems that deserve attention to be mitigated and promote quality of life and improve the health care provided to patients. Thus, we suggest developing strategies for the division of tasks between shifts to promote the health of these workers.

### Contributions to nursing, health, and public policies

This article contributes to the construction of nursing knowledge, as it addresses a current issue related to precarious work, working conditions, and shift work organization that can damage workers’ health. The awareness and understanding of implications of work on nursing professionals’ health can also help in the development of policies that improve working conditions and actions to prevent and promote workers’ health.

## CONCLUSION

From the perspective of local public health, the work carried out in a public hospital results in a higher prevalence of physical damage in nursing workers, especially in those who work the day shift. Willingness to be alone and headache showed a significant relationship with the day shift.

We need to develop research that investigates other work-related variables to identify relationships with workers’ health.

## SUPPLEMENTARY MATERIAL

The manuscript has research data available at https://doi.org/10.48331/scielodata.93EALF.
